# Use of co‐design methodology in the development of cardiovascular disease secondary prevention interventions: A scoping review

**DOI:** 10.1111/hex.13633

**Published:** 2022-11-10

**Authors:** Jason Talevski, Stefan T. Kulnik, Rebecca L. Jessup, Roman Falls, Natali Cvetanovska, Alison Beauchamp

**Affiliations:** ^1^ Institute for Physical Activity and Nutrition Research (IPAN), School of Exercise and Nutrition Sciences Deakin University Geelong Victoria Australia; ^2^ School of Rural Health Monash University Warragul Victoria Australia; ^3^ Australian Institute for Musculoskeletal Science (AIMSS) The University of Melbourne and Western Health St Albans Victoria Australia; ^4^ Ludwig Boltzmann Institute for Digital Health and Prevention Salzburg Austria; ^5^ Faculty of Health, Social Care and Education Kingston University and St George's University of London London UK; ^6^ Academic and Research Collaborative in Health La Trobe University Bundoora Victoria Australia; ^7^ Allied Health Research, Northern Health Epping Victoria Australia; ^8^ Western Centre for Health Research and Education, Sunshine Hospital St Albans Victoria Australia; ^9^ Office of Research, Northern Health Epping Victoria Australia; ^10^ Victorian Heart Institute Monash University Clayton Victoria Australia

**Keywords:** cardiovascular disease, co‐design, community‐based participatory research, consumers, secondary prevention, stakeholders

## Abstract

**Introduction:**

There is growing evidence to support the use of co‐design in developing interventions across many disciplines. This scoping review aims to examine how co‐design methodology has been used in the development of cardiovascular disease (CVD) secondary prevention interventions within health and community settings.

**Methods:**

We searched four academic databases for studies that used the co‐design approach to develop their intervention. Studies were included if consumers (adults with CVD) and key stakeholders (e.g. clinicians, service providers) were involved in the co‐design process. The review focused on methodology rather than traditional study outcomes; therefore, co‐design processes and activities were extracted and evaluated against a selected co‐design framework.

**Results:**

Twenty‐two studies were included in this review. Studies were implemented across various settings with consumers and stakeholder groups most frequently consisting of patients and healthcare professionals, respectively. Most studies specifically stated that they used a ‘co‐design’ approach (*n* = 10); others used terms such as participatory action research (*n* = 3), user‐centred design (*n* = 3) and community‐based participatory research (*n* = 2). Although there was variability in terminology, co‐design processes, and participants, all studies adhered to the key principles of consumer engagement. Predominant co‐design activities included semistructured interviews, focus groups, co‐design/development workshops and advisory group meetings. Intervention effectiveness was assessed in eight studies showing mixed results.

**Conclusions:**

This review provides an overview of how the co‐design approach has previously been used in the development of CVD secondary prevention interventions. These findings provide methodological considerations that can guide researchers and healthcare services when implementing co‐design to develop feasible and acceptable interventions that can improve outcomes for CVD populations.

**Patient or Public Contribution:**

No patients, service users, caregivers, people with lived experience or members of the public were involved in this scoping review. This review article was written by academics who have undertaken a significant amount of co‐design work with consumers and stakeholders.

## 1 INTRODUCTION

Cardiovascular diseases (CVDs) are a group of disorders of the heart and blood vessels and include coronary heart disease, stroke, heart failure and other conditions. CVD has remained the leading cause of death globally for the last 20 years and is estimated to cause more than 18 million deaths each year.^1^ Significant progress has been made over the past few decades in the management and treatment of CVD, particularly in the prevention of recurrent cardiovascular events in high‐risk individuals such as those with previous events or known CVD (i.e., secondary CVD prevention). Secondary prevention guidelines have been developed^2^, ^3^ that recommend lifestyle changes for ongoing management of cardiovascular risk factors, including a healthier diet (reduction of salt, eating more fruits and vegetables), regular physical activity, medications and cessation of tobacco use and harmful intake of alcohol. However, while these behaviour changes have been shown to significantly reduce the risk of secondary CVD events,^4^, ^5^, ^6^ they are also shown to be difficult to sustain long term.^7^, ^8^, ^9^

Several effective approaches to support CVD patients in adopting these preventive behaviours have been established, including engagement in lifestyle modification programmes, use of technology‐based interventions (e.g., telehealth, mobile health applications) and cardiac rehabilitation.^5^ However, despite good evidence for their effectiveness, these programmes are not always well‐utilized or accepted by the target population. For example, the benefits of cardiac rehabilitation are well‐recognized and include lower mortality rate, reduced risk of hospital admissions and improved health‐related quality of life.^10^, ^11^ However, cardiac rehabilitation continues to have poor attendance rates, with only 15%–30% of eligible patients engaging with the programme.^12^, ^13^ To address poor uptake, policymakers and healthcare providers need to ensure that interventions are well‐accepted and adapted to the specific needs and perceptions of the target population.^14^, ^15^ One way to achieve this is for researchers to work with end‐users and nonacademic stakeholders in the development of interventions; this is known as co‐design.

The engagement of stakeholders in the development of interventions or public health initiatives is captured in the literature under different terminologies such as ‘co‐design’, ‘co‐production’, ‘co‐creation’, ‘participatory action research’ or ‘user‐centred design’.^16^ These terms are often used interchangeably by authors and are understood to describe equivalent approaches to stakeholder engagement. Co‐design is a process in which targeted end‐users and other relevant stakeholders form a partnership with researchers and work together on all aspects of intervention development, from understanding the needs of end‐users to content development and pilot testing.^17^ There are a number of co‐design research frameworks in the literature, all describing a similar series of sequential phases and core principles, which include equity (shared decision‐making across all stages), understanding experiences (co‐learning with a mutual exchange of information between partners) and improving services or health outcomes (development of a programme based on the findings).^17^ Co‐design is a relatively new concept within healthcare, although over the past decade, researchers and healthcare providers have increasingly involved consumers and nonacademic stakeholders in the development of public health interventions to improve process and health outcomes.^18^ Involving consumers in this way has been shown to increase acceptance, uptake, long‐term adherence and satisfaction with interventions, as well as improve the health outcomes of end‐users.^19^, ^20^ For this reason, evidence for the use of the co‐design approach in the development of healthcare interventions is increasing across many disciplines.^21^ However, despite an abundance of CVD intervention studies in the literature, few describe the use of co‐design methodologies. Utilizing the co‐design approach could address potential barriers to the uptake of an intervention and may deliver more effective and sustainable solutions to CVD secondary prevention.^22^, ^23^

The aim of this review is to examine the nature and extent of co‐design methods utilized in the development of CVD secondary prevention interventions within health and community settings. Specific review questions are:

1. What *approaches/concepts* to co‐design have been used in CVD secondary prevention research (e.g., co‐production, participatory action research, etc.)?
2. What *activities/methods* were used to develop these CVD secondary interventions (e.g., focus groups, co‐design workshops, etc.)?
3. Have these co‐designed CVD interventions been evaluated for *effectiveness*, and if so, what health outcomes were evaluated?

By summarizing the key processes used to develop these CVD interventions, we intend to improve the knowledge base for co‐design in CVD research and provide a guide for researchers considering using these methods.

## 2 METHODS

A scoping review was undertaken to address our research questions following a methodological framework to guide the review process.^24^ The review is reported in accordance with the Preferred Reporting Items for Systematic reviews and Meta‐Analyses extension for Scoping Reviews (PRISMA‐ScR) Checklist^25^ and is registered with PROSPERO (CRD42021291841).

### 2.1 Search strategy

An electronic database search for published literature was performed in August 2022 using Ovid Medline (biomedical literature), Embase (biomedical and pharmaceutical literature), PsycINFO (psychology and behavioural sciences) and Web of Science (sciences, engineering, medicine and social sciences). A sensitive search strategy was first developed for Ovid Medline comprising a broad range of terms for ‘co‐design’ (Supporting Information: File 1) and then modified for other databases. Reference lists from eligible studies and systematic reviews discovered by the search were also reviewed for further relevant studies.

### 2.2 Eligibility criteria

#### 2.2.1 Study design and participants

All relevant English language original primary research studies utilizing qualitative, quantitative or mixed‐methods study designs were included. Systematic reviews/meta‐analyses, scoping and narrative reviews, opinion pieces, editorials, letters to the editor, government reports, conference abstracts and non‐English language publications were excluded. Studies were considered eligible for the review if consumers (adults aged ≥18 years with CVD including coronary heart disease, stroke, heart failure, heart attack/myocardial infarction or peripheral vascular disease), their carers and/or key stakeholders (clinicians, service providers and relevant stakeholder organizations) were involved in the co‐design process described by the study.

#### 2.2.2 Interventions

The definition of co‐design for this review was adopted from Boyd et al.^17^ that includes six core elements (Box 1). The first three elements aim to gain an understanding of the consumer experience and needs, while the latter three focus on how to improve that experience through development and action. Studies were included in this review if the intervention in question met the following three conditions: (1) steps in the development or design of the intervention were clearly described; (2) the methods used to develop the intervention matched our definition of co‐design (i.e., consumers and/or stakeholders were included in both the Exploratory and Development Phases of co‐design) and (3) the intervention aimed to improve management or prevention of subsequent cardiovascular events.

Box 1: **Elements of co‐design**

*Exploratory Phase*

1. Engage: Establish meaningful relationships with consumers and/or relevant stakeholders to understand and improve a problem.
2. Plan: Work with consumers and/or stakeholders to identify ideas about goals and how to achieve them.
3. Explore: Learn about consumers' and/or stakeholders' experiences and identify what can be improved (i.e., needs assessment).
*Development Phase*
4. Develop: Turn the ideas from consumers and/or stakeholders into potential solutions (i.e., intervention development).
5. Decide: Choose improvements to make and how to make them based on further feedback from consumers and/or stakeholders.
6. Change: Turn improvement ideas into actions (e.g., prototype testing with end‐users) and finalize the intervention.

#### 2.2.3 Outcomes

The main outcomes of interest were the methods and phases of the co‐design process used in each study. This included the number and type of participants involved (consumers and key stakeholders), the degree of consumer and/or stakeholder involvement, and intervention feasibility/acceptability. If reported, data on the evaluation of intervention effectiveness were also collected.

### 2.3 Study selection

Two authors (J. T. and A. B.) screened abstracts of potentially relevant studies against the eligibility criteria using Covidence—a web‐based platform for undertaking the steps in the study review process.^26^ Of the potentially relevant studies from this initial screening, full‐text articles were obtained and assessed for inclusion independently by two authors (J. T. and one other author). If there were any conflicts between reviewers, a third author was called upon to make the final decision.

### 2.4 Data charting

The following data were independently extracted from each study by two authors (J. T. and A. B.): lead author, publication year, country of study, study design, consumer characteristics (number, % male, mean age), stakeholder characteristics (number, % male, mean age), intervention description, methods of co‐design and outcome data (feasibility, acceptability and effectiveness). If any discrepancies were observed between the extracted data, the authors met to reach a final consensus.

### 2.5 Critical appraisal of evidence

As this was a scoping review, a formal assessment of methodological quality was not undertaken. However, to meet the aims of the review, a sufficiency of the reporting approach was undertaken using an amended version of the Critical Appraisal Skills Programme (CASP) critical appraisal tools designed for multiple research study designs.^27^ The items included:

1. Aim (was there a clear statement of the aims of the research?)
2. Setting (was it clear where the development of the intervention took place?)
3. Recruitment (was it clear how the study participants were recruited?)
4. Participants (was it clear which consumers/stakeholders were involved in the co‐design process, and do you know all that you need to about the participants?)
5. Facilitators (was it clear who facilitated the co‐design process?)
6. Procedure (was the description of the overall co‐design process clear/complete?)
7. Schedule (were the interval and frequency of the co‐design sessions clear?)
8. Results (were the results of the study clearly documented and discussed?)
9. Intervention (was the final version of the intervention clearly described?)

### 2.6 Synthesis of results

A co‐design methodology assessment tool was developed based on Boyd et al.'s^17^ co‐design framework. To determine how co‐design has been previously implemented in CVD populations, included studies were assessed for their inclusion of Boyd et al.'s^17^ six elements of co‐design framework: engage, plan, explore, develop, decide and change. The ‘develop’ and ‘decide’ steps were reported together in the results as the synthesis found these steps coincided with each other within all studies. Where available, data of effectiveness were summarized thematically. Data synthesis was completed by the first author (J. T.) and checked for consistency by one other author (A. B.).

## 3 RESULTS

### 3.1 Study selection

**Figure 1:**
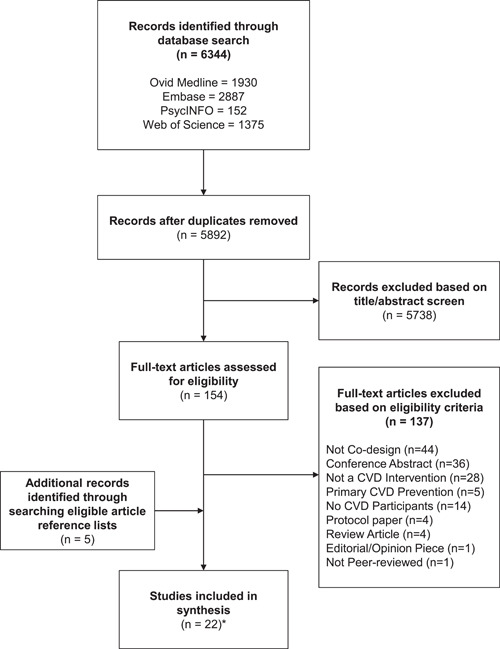
Flowchart of study selection. *Some studies published their methods and results across multiple papers, and as such, a total of 45 articles were retrieved that contributed to data synthesis in this scoping review.

Following the removal of duplicates, 5892 articles were retrieved from the electronic search. Of these, 5738 articles were excluded based on the review of the title and abstract, and the full text was obtained for the remaining 154 articles. Based on the authors' assessment, 22 unique studies^28^, ^29^, ^30^, ^31^, ^32^, ^33^, ^34^, ^35^, ^36^, ^37^, ^38^, ^39^, ^40^, ^41^, ^42^, ^43^, ^44^, ^45^, ^46^, ^47^, ^48^, ^49^ published across 45 articles met the eligibility criteria and were included in this review (Figure 1).

### 3.2 Study and participant characteristics

Of the 22 included studies, 16 utilized a mixed‐method approach^28^, ^29^, ^30^, ^32^, ^34^, ^35^, ^38^, ^39^, ^41^, ^42^, ^43^, ^44^, ^45^, ^47^, ^48^, ^49^ and six were qualitative.^31^, ^33^, ^36^, ^37^, ^40^, ^46^ Only one study had been published before the year 2010,^39^ while the rest were published between 2014 and 2022. Multiple geographical settings were represented with the majority of studies from Australia (*n* = 7) and the United States (*n* = 4), and two studies each from the Netherlands, Sweden and the United Kingdom. Studies were implemented across various settings including hospitals, outpatient clinics, primary care centres and community centres/organizations.

All 22 studies reported the number and type of participants involved in the co‐design process, although demographic information such as age, gender and socioeconomic position of consumers and stakeholders were poorly reported. Almost all of the studies included both consumers and other key stakeholders in their co‐design methodology, with the exception of one study^29^ that only involved consumers. Consumer perspectives were represented by intervention end‐users including patients, caregivers and/or family members (range: 9–178), while key stakeholder groups mostly consisted of healthcare professionals (nurses, doctors and allied health professionals), hospital management staff or representatives from key stakeholder organizations (range: 6–282). Table 1 presents a summary of the study and participant characteristics.

**Table 1:** Study and participant characteristics

References	Country	Study setting	Study design	Consumer involvement		Stakeholder involvement	
				Participants	*N*	Participants	*N*
Aaby et al.^28^	Denmark	Outpatient	Mixed‐methods	CR participants	178	CR staff and CR team leaders	35
Ahmed et al.^29^	USA	Outpatient	Mixed‐methods	HF patients and caregivers	43	‐	**‐**
Bonner et al.^30^	Australia	Primary care	Mixed‐methods	CVD patients and people with ≥1 CVD risk factor	34	GPs	282
Breeman et al.^31^	Netherlands	Outpatient/community	Qualitative	CVD patients, patient representatives and CR participants	35	Nurses, cardiologists, physical therapists, GPs, psychologists, neurologists, lifestyle coaches	58
Cornet et al.^32^	USA	Outpatient	Mixed‐methods	HF patients and family/friends	73	Cardiologists, clinic supervisors and managers, technicians, nurses	7
Dorri et al.^33^	Iran	Hospital	Qualitative	ACS patients and family members	38	Nurses, faculty members, cardiologists, educational supervisors	10
Driver et al.^34^	USA	Hospital	Mixed‐methods	Stroke patients and carers	32	Physiotherapists, occupational therapists, speech therapists, exercise specialists, dieticians, a stakeholder organization representative	22
Hjelmfors et al.^35^	Sweden	Outpatient	Mixed‐methods	HF patients and family members	11	Physicians and nurses	25
Kjork et al.^47^	Sweden	Outpatient/community	Mixed‐methods	Stroke patients	22	Nurses, occupational therapists, physicians, physiotherapists, neuropsychologists, speech therapists	11
Lalonde et al.^36^	Canada	Primary care	Qualitative	CVD patients and family members	20	Physicians, pharmacists, nurses, nutritionists, psychologists	52
Pekmezaris et al.^37^	USA	Community	Qualitative	Patients, caregivers and patient advocates	10	Cardiologists, geriatricians, nurses, pharmacists, health policy workers	8
Prick et al.^48^	Netherlands	Hospital	Mixed‐methods	Stroke patients and carers	77	Neurologists, rehabilitation specialists, geriatricians, nurses, occupational therapists, physiotherapists, speech therapists	111
Ramage et al.^49^	Australia	Community	Mixed‐methods	Stroke patients and carers	16	Healthcare workers	21
Raynor et al.^38^	UK	Hospital/primary care	Mixed‐methods	HF patients and carers/family members	75	GPs, heart failure nurses, heart failure consultants, pharmacists, hospital managers	103
Redfern et al.^39^	Australia	Outpatient	Mixed‐methods	Angina/MI patients and people with ≥1 CVD risk factor	52	Allied health professionals, medical staff, cardiologists, nurses	6
Sabater‐Hernandez et al.^40^	Australia	Community	Qualitative	AF patients and patients with hypertension	9	Pharmacists, GPs, cardiologists, cardiac/research nurses, a stakeholder organization representative	11
Toledo‐Chavarri et al.^41^	Spain	Community	Mixed‐methods	IHD Patients	25	GPs, nurses, cardiologists	10
Tongpeth et al.^42^	Australia	Hospital/community	Mixed‐methods	ACS patients	20	Cardiologists, cardiac nurses, cardiac researchers	12
Triantafyllidis et al.^43^	UK	Hospital/community	Mixed‐methods	HF patients and caregivers	78	Cardiologists, nurses, GPs, hospital administrators	23
Walsh et al.^44^	Ireland and Belgium	Hospital	Mixed‐methods	CVD Patients	72	Community, representatives, GPs, nurses, cardiologists, CR coordinators	31
Woods et al.^45^	Australia	Hospital/community	Mixed‐methods	HF patients and carers	13	Nurses, cardiologists, allied health professionals	20
Zacharia et al.^46^	Australia	Outpatient/community	Qualitative	Stroke patients and carers	14	Stroke rehabilitation clinicians, dietitians	15

Abbreviations: ACS, acute coronary syndrome; AF, atrial fibrillation; CR, cardiac rehabilitation; CVD, cardiovascular disease; GP, general practitioner; HF, heart failure; IHD, ischaemic heart disease; UK, United Kingdom; USA, United States of America.

### 3.3 Intervention characteristics

The interventions developed across studies were broad, but all aimed to improve the management of CVD or prevent further cardiovascular events. Five interventions focused on the broad area of CVD secondary prevention, whereas other interventions focused on specific cardiac disorders such as heart failure (*n* = 7), stroke (*n* = 5), acute coronary syndrome (*n* = 2), coronary heart disease (*n* = 1), atrial fibrillation (*n* = 1) and ischaemic heart disease (*n* = 1). Fourteen studies described the development of an e‐health intervention such as health data dashboards or online platforms,^29^, ^30^, ^31^, ^41^, ^44^, ^47^, ^48^ mobile/tablet applications^32^, ^42^, ^43^, ^45^ and telehealth programmes.^37^, ^46^, ^49^ Two interventions aimed to improve or address the health literacy of patients,^28^, ^41^ three focused on improving patient self‐empowerment and behaviour change,^34^, ^41^, ^49^ and there were two exercise‐based interventions.^44^, ^49^ Almost all interventions (*n* = 20) included patient education on the importance of healthy behaviours for cardiovascular health or information on self‐management of CVD risk factors (medications, exercise, diet, etc.). The interventions of the included studies are described in detail in Supporting Information: File 2.

### 3.4 Co‐design methodology

Although study authors used various terminology and described different definitions of co‐design research, all the included studies adhered to the principles of consumer engagement, where consumers and relevant stakeholders formed a partnership with researchers and took an active role in intervention development. Most of the studies stated that they used a ‘co‐design’ approach (10 studies), while other studies identified their approach as participatory action research (3 studies), user‐centred design (3 studies), community‐based participatory research (2 studies), participatory research (2 studies) or co‐production (*n* = 1). One study did not report using co‐design methodology but instead described their approach as ‘gaining patient and healthcare professional input’.^39^ Many different roles for participants were also described. This varied from advisors and committee members who provided advice on co‐design methodology, reviewers who examined and assessed the implications of findings from co‐design tasks, and co‐designer roles where the participants were integrally involved in intervention development. Research methods and co‐design activities varied across studies: the predominant co‐design activities conducted with consumers and stakeholders across studies were focus groups (14 studies), semistructured interviews (13 studies), co‐design/development workshops (11 studies), advisory group/working group meetings (7 studies) and ideas/brainstorming meetings (5 studies). Eight studies tested the intervention in a ‘real world’ feasibility study that provided feedback on usability, acceptability or suitability. Table 2 details the co‐design methodologies utilized in the included studies.

**Table 2:** Co‐design methodology of included studies

References	Co‐design methodology		Boyd et al.^17^ elements of co‐design					
	Approach^a^	Methods/activities	Engage	Plan	Explore	Develop	Decide	Change
Aaby et al.^28^	Co‐design	User health literacy assessments; Semistructured interviews; Focus groups; Ideas workshops	**X**	**✓**	**✓**	**✓**	**✓**	**✓**
Ahmed et al.^29^	Participatory research	Focus groups; Design workshops; Feasibility study	**X**	**X**	**✓**	**✓**	**✓**	**✓**
Bonner et al.^30^	Co‐design	‘Think aloud’ interviews; Semistructured interviews; Design workshops; Feasibility study	**X**	**✓**	**✓**	**✓**	**✓**	**✓**
Breeman et al.^31^	User‐centred design	Semistructured interviews; Usability workshop; Focus groups; Stakeholder workshop; ‘Think aloud’ interviews	**X**	**✓**	**✓**	**✓**	**✓**	**✓**
Cornet et al.^32^	User‐centred design	Critical incident interviews; Scenario‐based cognitive interviews; Usability evaluation workshops; Advisory group meetings	**X**	**X**	**✓**	**✓**	**✓**	**✓**
Dorri et al.^33^	Participatory action research	Brainstorming meetings; Semistructured interviews; Focus groups	**✓**	**✓**	**✓**	**✓**	**✓**	**✓**
Driver et al.^34^	Community‐based participatory research	Focus groups; Advisory board meetings	**✓**	**✓**	**✓**	**✓**	**✓**	**✓**
Hjelmfors et al.^35^	Co‐design	Ideas workshops; Feasibility study	**X**	**✓**	**✓**	**✓**	**✓**	**✓**
Kjork et al.^47^	Co‐design	Expert panel meetings; Focus groups; Interviews; Surveys; Co‐design workshops	**✓**	**✓**	**✓**	**✓**	**✓**	**✓**
Lalonde et al.^36^	Participatory research	Needs assessment workshop; Focus groups; Appropriateness surveys; Working‐group meetings; Semistructured interviews	**X**	**✓**	**✓**	**✓**	**✓**	**X**
Pekmezaris et al.^37^	Community‐based participatory research	Focus groups; Co‐design workshops	**✓**	**✓**	**✓**	**✓**	**✓**	**✓**
Prick et al.^48^	User‐centred design	Steering group meetings; Surveys; Focus groups; Co‐creation workshops; Think‐aloud interviews	**X**	**✓**	**✓**	**✓**	**✓**	**✓**
Ramage et al.^49^	Co‐production	Co‐production team meetings; Ideas workshops; Development workshops; Interviews	**✓**	**✓**	**✓**	**✓**	**✓**	**✓**
Raynor et al.^38^	Co‐design (experience‐based)	Hospital ward observations; Semistructured interviews; Co‐design meetings; Feasibility study	**✓**	**✓**	**✓**	**✓**	**✓**	**✓**
Redfern et al.^39^	‐	Semistructured interviews; Readability and suitability questionnaires	**X**	**X**	**✓**	**✓**	**✓**	**✓**
Sabater‐Hernandez et al.^40^	Co‐design	Focus groups; Semistructured interviews	**X**	**X**	**✓**	**✓**	**✓**	**✓**
Toledo‐Chavarri et al.^41^	Co‐design	Listening labs; Focus groups; Development workshops; Online feasibility activities	**X**	**✓**	**✓**	**✓**	**✓**	**✓**
Tongpeth et al.^42^	Participatory action research	Focus groups; Co‐design workshops; Feasibility study	**X**	**✓**	**✓**	**✓**	**✓**	**✓**
Triantafyllidis et al.^43^	Participatory action research	Semistructured interviews; Co‐design workshops; Feasibility study	**X**	**✓**	**✓**	**✓**	**✓**	**✓**
Walsh et al.^44^	Co‐design	Stakeholder expert panel; Semistructured interviews; Focus groups; Co‐design workshops; Usability testing workshops and questionnaires	**X**	**✓**	**✓**	**✓**	**✓**	**✓**
Woods et al.^45^	Co‐design	Semistructured interviews; Co‐design workshops; Development focus groups; Feasibility study	**X**	**✓**	**✓**	**✓**	**✓**	**✓**
Zacharia et al.^46^	Co‐design	Collaboration meetings; Co‐design workshops	**✓**	**✓**	**✓**	**✓**	**✓**	**✓**

^a^ As stated by the study authors.

#### 3.4.1 Step 1: Engage

Seven studies reported establishing meaningful relationships with consumers and stakeholders, which was the least reported co‐design process utilized across the included studies (Table 3). Five studies^34^, ^37^, ^46^, ^47^, ^49^ developed a type of community advisory board or expert panel comprised of patients, healthcare professionals and key stakeholders that either defined the research questions, devised notions to engage participants in the co‐design process or participated in the co‐design process to identify the essential elements of the intervention. One study undertaken in a hospital setting^38^ involved meeting with hospital management staff to identify key services and/or staff who could act as champions for the co‐design process. One study^33^ engaged nurses and nurse managers of the cardiac care unit via several informal meetings throughout the co‐design process to identify ways to address readmissions.

**Table 3:** Summary of co‐design elements

Phase	Element	Number of studies including phase (%)
Exploratory Phase	Engage	7 (32)
	Plan	18 (82)
	Explore	22 (100)
Development Phase	Develop	22 (100)
	Decide	22 (100)
	Change	21 (95)
Implementation Phase^a^	Evaluation	10 (45)

^a^ This phase is not part of Boyd et al.'s^17^ framework for co‐design.

#### 3.4.2 Step 2: Plan

Eighteen studies reported working with consumers and/or stakeholders to generate ideas about study goals and how to achieve them. The most common approach used in the planning phase was organizing stakeholder/expert or community advisory board meetings utilized in eight studies.^31^, ^37^, ^38^, ^42^, ^46^, ^47^, ^48^, ^49^ Group discussions/focus groups with participants were used in six studies^30^, ^34^, ^36^, ^41^, ^43^, ^44^; and four studies^28^, ^33^, ^35^, ^45^ ran ideas/brainstorming workshops to generate ideas/goals and devise plans on how to achieve them.

#### 3.4.3 Step 3: Explore

All 22 studies reported learning about consumers' or stakeholders' experiences with CVD to identify the challenges and priorities to improve CVD secondary prevention. Most studies undertook focus groups or interviews with consumers to better understand patient needs for healthcare or knowledge/awareness of cardiovascular health, or interviews with healthcare professionals to explore their experiences of working in cardiac healthcare services or to discover professionals' needs regarding helping patients with CVD. Only two studies used quantitative approaches in their need assessment by means of a cross‐sectional survey analysis.^28^, ^48^

#### 3.4.4 Steps 4–5: Develop and decide

All 22 studies included an intervention development phase where ideas from consumers and stakeholders were discussed and turned into potential solutions. These steps were described differently across all studies (focus groups; co‐design workshops; development workshops; advisory group meetings and working group meetings); however, generally included consumers and stakeholders coming together multiple times to discuss intervention ideas based on the needs of consumers, prioritize ideas for interventions and design an intervention prototype(s).

#### 3.4.5 Step 6: Change

Twenty‐one studies conducted pretesting of intervention prototypes with end‐users to gather further feedback on core attributes of the intervention design and to assess the acceptability and usability of the intervention. The majority of studies (*n* = 17) utilized a mixed‐method approach in gathering feasibility data, while the other four studies used qualitative approaches such as design workshops to elicit feedback before the intervention was finalized.

### 3.5 Intervention effectiveness

Intervention effectiveness was assessed in 10 studies^30^, ^33^, ^34^, ^35^, ^42^, ^43^, ^44^, ^46^, ^48^, ^49^ showing mixed results (Supporting Information: File 2). In a pre‐post feasibility study with 98 GPs, Bonner et al.^30^ found that the use of a newly designed CVD online platform increased the capacity for GPs to correctly identify the CVD risk categories of their patients (16% for low‐risk cases, 32% for moderate‐risk cases and 50% for high‐risk cases). The educational intervention developed by Hjelmfors et al.^35^ improved the self‐reported knowledge of heart failure, confidence and skills of 13 cardiac nurses (patients were not included in the evaluation). Dorri et al.^33^ also employed a pre‐post study to evaluate a nurse‐led, hospital‐based intervention in 31 acute coronary syndrome patients, which resulted in hospital readmission rates reducing from 32.2% to 12% at 6 months. Three studies^42^, ^43^, ^44^ conducted a randomized controlled trial to test the intervention created by the co‐design process. The patient education Avatar app developed by Tongpeth et al.^42^ was found to significantly improve knowledge, attitudes and beliefs of heart attack symptoms in intervention participants compared to controls at a 6‐month follow‐up (*n* = 70 patients), but no significant differences between groups were observed in healthcare utilization (GP visits, ED visits or 30‐day readmissions). In a randomized trial with 202 heart failure patients that tested the efficacy of a digital home monitoring system with an integrated risk prediction and disease management service, Triantafyllidis et al.,^43^ reported no statistically significant between‐group differences in treatment opportunity or health‐related quality of life at a 6‐month follow‐up. The randomized controlled trial by Walsh et al.^44^ evaluated an e‐health intervention for the self‐management of CVD risk factors in 120 CVD patients and found an increase in moderate to vigorous physical activities and a stable CVD risk score in intervention participants compared to controls at a 6‐month follow‐up. However, there were no significant differences between groups regarding most physical fitness outcomes, health‐related quality of life, mental health, exercise self‐efficacy, medication adherence or diet. Four studies^34^, ^46^, ^48^, ^49^ reported commencing a randomized controlled trial, and therefore, results were not available at the time of undertaking this review.

### 3.6 Sufficiency of reporting

The median score of the sufficiency of reporting using our amended CASP checklist was seven out of a maximum score of nine (range: 4–9), indicating that most studies described their co‐design methodology adequately. Authors of all studies reported the aims of the project clearly and the majority of studies reported the study results, including the design of the final intervention, and co‐design study schedule/procedures. Common omissions of information identified across studies included not reporting the study setting clearly and inadequately describing the characteristics of participants or how they were recruited (Supporting Information: File 3).

## 4 DISCUSSION

This is the first review on the use of co‐design methodology in the development of CVD secondary prevention interventions. Our findings confirmed that the co‐design approach is still an emerging field in CVD research, as evidenced by the small number of studies found in the literature with the majority of these published in the past 5 years. Studies were implemented across various settings with consumers and stakeholder groups most frequently consisting of patients and healthcare professionals, respectively. Consistent with co‐design literature,^50^ varying models of co‐design methodology utilizing different research activities were reported by studies. Findings from this review suggest it is feasible to apply the principles of co‐design in various settings and CVD population groups.

The lack of a singular consistent definition of ‘co‐design’ made it difficult to retrieve relevant literature for this review. Many research co‐design approaches were identified within the literature, with extensive variability in the methods, research phases, participants and levels of involvement. Based on the sufficiency of reporting checklist, most studies in this review described their co‐design methodology clearly and adequately, making it easier for others to replicate co‐design in their own research. Conversely, previous reviews of co‐design have reported that studies provided insufficient details of their co‐design activities in their methods to establish what was actually involved.^18^, ^50^, ^51^ This difference in reporting may be explained by our comprehensive inclusion criteria to draw together the varying definitions and terminology used, which was guided by a co‐design framework.^17^ During the full‐text review stage of the literature search, study methodology was evaluated against Boyd et al.'s^17^ co‐design framework to determine if the intervention was developed using established co‐design methodology; that is, consumers and/or relevant stakeholders were involved in most aspects of intervention development, from the needs assessment through to content development and pilot testing. Over 40 studies were excluded based on this definition, including 6 studies that specifically stated they used a co‐design or participatory action research approach. After closer inspection of these six studies, consumers and stakeholders either participated only in a need assessment to guide researchers in the development of the intervention (Exploratory Phase) or were asked to provide feedback on an intervention that was already developed by the researchers (Development Phase). For the purposes of this review, this was not considered a ‘true’ co‐design approach. The heterogeneity in approaches, variable use of terminology and gaps in reporting indicate that this area of research may benefit from a framework that sets out the core principles for cardiac services seeking to use co‐design.

Engagement with consumers and stakeholders is critical to true and successful co‐design.^17^ Although all 22 studies reported learning about consumers' or stakeholders' experiences with CVD (i.e., performed a needs assessment) and included them in the development phase of the intervention, few studies reported methods of establishing meaningful relationships with consumers and stakeholders. This involved strategies such as developing a community advisory board comprised of key stakeholders, identifying local champions to oversee the study, and engaging with key staff before beginning the research to help guide the co‐design process. Previous studies have identified that selecting stakeholders strategically to fit project needs, adapting the project to the practical needs of stakeholders and clearly defining roles and expectations for stakeholders, as well as responsibilities and powers, are key strategies to establishing meaningful partnerships in research.^52^ Broad engagement principles such as these need to be present when using co‐design methods to develop effective and participatory research relationships, rather than engaging stakeholders merely to fulfill a requirement. Additionally, having stakeholders involved early means that their experiences and requirements can be taken into account at the start of the process rather than researchers presuming to know what is required.^53^ Researchers and policymakers who are new to the co‐design approach should consider how involving end‐users and stakeholders in the study planning phase can assist in prioritizing research topics, setting research agendas and helping to refine research design and processes.

Assessing the effectiveness of co‐designed interventions in formal evaluations and clinical trials is important to determine the efficacy of this method.^18^ There is minimal literature that has evaluated the direct links between co‐designed CVD interventions and improved patient experiences or outcomes. In this review, intervention effectiveness was only assessed in 10 studies and mixed results were reported across studies. While few studies to date have investigated the effectiveness of co‐designed interventions in CVD secondary prevention, a recent systematic review and meta‐analysis found that co‐designed public health interventions for various health conditions can improve short‐term health outcomes such as self‐efficacy, healthy behaviours and lifestyle changes, health service access and physical health.^54^ Further to this, the majority of studies included in this review can be considered complex interventions (i.e., having multiple interacting components or target groups and settings).^55^ An important element of complex interventions is understanding ‘how’ and under what circumstances an intervention can achieve its desired effect, often termed the ‘mechanism of change’. These mechanisms, or causal links, are more transparent when ‘theory’ is applied to the development of an intervention, also facilitating meaningful evaluation of outcomes.^55^ Of the included studies, only seven applied a theoretical framework during the intervention development. These included the Behaviour Change Wheel,^30^, ^46^ the Social Cognitive Theory,^44^ the Medical Research Council (MRC) framework,^38^ the Ophelia approach^28^ and the Integrated Knowledge Translation theoretical approach.^46^, ^49^ However, the way in which these theories informed evaluation was not clearly articulated. In other studies, authors proposed suggestions for mechanisms of change, such as tailoring interventions to the needs of end‐users,^35^, ^40^, ^41^, ^42^ but these were not underpinned by a theoretical framework. To advance robust reporting of co‐designed interventions, authors are encouraged to consider the mechanisms of action in both the design and evaluation of their interventions and to explicitly describe these to readers using public health frameworks such as the Template for Intervention Description and Replication (TIDieR) checklist.^56^

The issue of implementation is increasingly understood to be an integral point of attention throughout the course of intervention development, from conceptualization to evaluation. During the pilot testing/evaluation phase, aspects of implementation are typically addressed through process evaluations,^57^ and more recently, through innovative hybrid effectiveness‐implementation study designs that provide outcomes on both intervention effectiveness and implementation (e.g., implementation fidelity, the proportion of reach and barriers to implementation).^58^ In contrast, issues of implementation are generally addressed within the stages of the co‐design approach through dedicated stakeholder engagement. The MRC framework for complex interventions highlights the value of stakeholder engagement throughout all stages of intervention development and evaluation: prioritizing research questions, development of the programme theory, intervention refinement and practical support for evaluation.^55^ Engagement of stakeholders can also help to clarify which contextual factors might influence both the implementation and effectiveness of an intervention.^59^ Understanding the role of these contextual factors is particularly important when considering scalability (i.e., implementing a locally designed intervention in multiple settings where operating systems and delivery of care are likely to vary).^55^ Involving stakeholders who hold relevant, lived experience and including a dedicated focus on implementation in the co‐design process may support successful upscaling and dissemination of interventions to a wide range of settings.

Although co‐design has been used frequently to develop health interventions for ‘underserved’ population groups such as people with disabilities or from culturally and linguistically diverse communities,^60^, ^62^ it was interesting to find that only one study involving these populations was identified in this review. Pekmezaris et al.^37^ included Black and Hispanic patients and disparity experts in their study to create a telemonitoring intervention for heart failure that is acceptable and feasible for use with a lower‐income, Black and Hispanic patient population. More studies in this review may have included participants representing diverse groups; however, our sufficiency of reporting analysis found that studies often lacked an adequate description of participant characteristics. The burden of CVD is higher among minority groups and people living with socioeconomic disadvantage, which and is associated with increased mortality rates and a greater risk of subsequent cardiac events.^63^ Evidence also shows that these populations receive suboptimal healthcare access compared to other population groups.^64^ Healthcare services have an important role to play in addressing this social gradient,^65^ by ensuring that the care they provide is accessible for all, including culturally and linguistically diverse communities, minority groups and those with lower socioeconomic status.^66^ Use of the co‐design approach can take context‐specific challenges of ‘underserved’ population groups into consideration to provide a culturally appropriate and logistically sound CVD intervention that is feasible and acceptable.

### 4.1 Strengths and limitations

The strengths of this review include were our rigorous methodology and comprehensive search strategy. We have confidence that we identified all published studies that met our inclusion criteria as we used various synonyms of ‘co‐design’ in our search strategy. Furthermore, we excluded studies that were assessed as not genuinely utilizing the co‐design approach so we could accurately examine the use of co‐design in CVD secondary prevention research. By summarizing the co‐design processes across multiple studies, we have improved the knowledge base for co‐design in CVD research and provided a guide for future CVD researchers considering using these methods. Limitations of our review should also be considered. Searches were limited to published studies, subjecting this review to the possibility of publication bias. Further to this, because we restricted our search to studies published in English, we may have omitted relevant research written in other languages. It would have been useful to understand the link between specific co‐design methods and research outcomes; however, this was not possible due to the heterogeneity of co‐design activities in the included studies, although determining intervention effectiveness was not the primary aim of this review. Lastly, no consumers (i.e., people with lived experience) or stakeholders were involved in undertaking this scoping review, which may have supported a more critical interpretation of the implementation of co‐design approaches in the current CVD literature. However, as this was an unfunded review project, we had no resources to conduct consumer involvement activities such as offering adequate education and training (scoping reviews require research knowledge or training that consumers commonly do not hold) and remuneration to consumers for their time.

## 5 CONCLUSION

We conducted a scoping review to examine how studies have undertaken co‐design to develop CVD secondary prevention interventions. In doing so, we make several practical contributions to the literature. First, the findings from this review provide methodological considerations that can guide researchers and healthcare services in understanding how the co‐design approach may be implemented to develop locally feasible, acceptable and sustainable strategies to improve outcomes for CVD populations. Second, by adapting Boyd et al.'s^17^ co‐design framework, we mapped the co‐design activities used in studies to the six steps of co‐design to ensure sufficient reporting of methods for future research. Finally, we identified the knowledge gaps in CVD co‐design research (e.g., limited research in post‐design evaluation, inadequate reporting of levels of consumer involvement), highlighting the importance of assessing the effectiveness of co‐designed interventions in future research.

## AUTHOR CONTRIBUTIONS

The study was conceived by Jason Talevski and Alison Beauchamp. Jason Talevski conducted the literature search and all authors contributed to the study selection. Jason Talevski and Alison Beauchamp extracted, analysed and interpreted the data. Jason Talevski, Roman Falls, Natali Cvetanovska and Alison Beauchamp conducted the critical appraisal of studies. Jason Talevski drafted the manuscript, and all authors critically revised the manuscript and approved the final version.

## CONFLICT OF INTEREST

The authors declare no conflict of interest.

## Supporting information

Supplementary information.

Supplementary information.

Supplementary information.

## Data Availability

No new data were generated or analysed in support of this research.
