# Supplementary material for: Use of co‐design methodology in the development of cardiovascular disease secondary prevention interventions: A scoping review
Source: Health Expect. 2022 Nov 10;26(1):16–29. doi: 10.1111/hex.13633 (PMC9854329; doi:10.1111/hex.13633)
Supplement: Supplementary file 1 — Supplementary information. [file HEX-26--s003.docx]

**Supplementary File 1: Search strategy for Ovid MEDLINE**

| **#** | **Search Term(s)** |
| --- | --- |
| 1 | exp Cardiovascular Diseases/ |
| 2 | exp Heart/ |
| 3 | exp Coronary Disease/ |
| 4 | exp Coronary Artery Disease/ |
| 5 | exp Myocardial Infarction/ |
| 6 | exp Acute Coronary Syndrome/ |
| 7 | 1 or 2 or 3 or 4 or 5 or 6 |
| 8 | Co-design.mp. |
| 9 | Codesign.mp. |
| 10 | Co-produc*.mp. |
| 11 | Coproduc*.mp. |
| 12 | Co-creat*.mp. |
| 13 | Cocreat*.mp. |
| 14 | exp Community Participation/ |
| 15 | exp Community-Based Participatory Research/ |
| 16 | exp Stakeholder Participation/ |
| 17 | 8 or 9 or 10 or 11 or 12 or 13 or 14 or 15 or 16 |
| 18 | 7 and 17 |
| 19 | limit 18 to (humans and yr="1990 - Current") |
