# Supplementary material for: Use of co‐design methodology in the development of cardiovascular disease secondary prevention interventions: A scoping review
Source: Health Expect. 2022 Nov 10;26(1):16–29. doi: 10.1111/hex.13633 (PMC9854329; doi:10.1111/hex.13633)
Supplement: Supplementary file 2 — Supplementary information. [file HEX-26--s001.docx]

**Supplementary File 2:** Intervention characteristics of and evaluation of effectiveness results

| **Author (year)** |  | **Intervention characteristics** | | |  | **Evaluation of effectiveness** | | |
| --- | --- | --- | --- | --- | --- | --- | --- | --- |
|  |  | **Population/ condition** | **Setting** | **Description** |  | **Design** | **Participants** | **Key Findings** |
| Aaby et al. (2020) |  | CVD | Hospital & CR clinic | *Intervention 1:* A health literacy intervention to improve the social support of people referred to CR: 1) handing out written information in hospital to support relatives/friends; 2) verbally inviting patients to bring a relative/friend to their first CR session; and 3) offering a voluntary ‘relative’ when a referred person has no one to bring.  *Intervention 2:* A health literacy intervention to identify and respond to the needs of vulnerable people referred to the CR: 1) identifying vulnerability based on an assessment of health literacy; 2) identifying mental challenges or ‘at risk of non-adherence’ to the CR; and 3) offering an extra needs identification and program planning session to vulnerable groups, leading to an individualized care plan. |  | - | - | - |
| Ahmed et al. (2019) |  | Heart Failure | Community (online) | A heart failure health data dashboard for patients to help visualise key data from an implantable cardiac resynchronisation therapy device. Patients receive verbal instructions and a take-home packet from a research nurse on accessing and navigating the dashboard, as well as the actions to take if in a yellow zone (watch for symptoms indicative of worsening condition) or red zone (contact clinic). Patients could access their dashboard at any time for 6-12 months. |  | - | - | - |
| Bonner et al. (2019) |  | CVD | GP Clinic | An online platform for GPs to use with their patients in consultations, which includes the following features: 1) a summary of Australian guidelines for CVD risk assessment and management for low, moderate and high risk patients; 2) updated evidence summaries based on rapid reviews for high quality studies on lifestyle and medication interventions; 3) an interactive CVD risk calculator; 4) a personalized patient decision aid that shows the effect of different medication, lifestyle (smoking, diet, exercise) and supplement interventions on individual CVD risk to help GPs determine different treatment options; and 5) a self-directed audit & feedback form including cases that GPs find challenging for CVD risk assessment and communication, and comparison of management to guidelines. |  | Pre-post study | 98 GPs | Increase in identification of correct CVD risk category for low-risk cases by 16%, moderate-risk cases by 32% & high-risk cases by 50% |
| Breeman et al. (2021) |  | CVD | Community  (online) | An e-health digital platform that provides an overview of interventions and initiatives to support healthy living in the context of CVD prevention and rehabilitation. The digital platform includes a number of core attributes including automatic exchange of data between health information systems and interventions; education and skills training; goal setting and revision; action planning; monitoring health and behaviour; feedback on progress; praise and reminder messages; a rewards program; providing social support and stimulating intrinsic motivation; and offering continuity of care via chat, phone, and/or video counselling. |  | - | - | - |
| Cornet et al. (2019) |  | Heart Failure | Community  (smartphone app) | *Power to the Patient (P2P)* is a mobile application which aims to provide better and timelier self-care among heart failure patients with a cardiac implantable electronic device. P2P helps patients react to risk of heart failure events as detected by their devices, thus preventing unnecessary hospitalizations. P2P also collects self-assessment on the four heart failure self-care domains of medication self-administration, dietary sodium intake, fluid intake, and physical activity; and recommends self-care activities and practical strategies based on user self-assessments in each domain. |  | - | - | - |
| Dorri et al. (2021) |  | ACS | Hospital | Establishing a discharge monitoring team, allocating a mobile number from 4:00PM to 8:00PM to track patients and to get advice from a nurse, developing a free and 24‑hour telephone line for patients to contact nurses, and implementing face‑to‑face CVD education for each patient during admission and right before being discharged. |  | Pre-post study | 31 ACS patients | Hospital readmission rate at 6-months went from 32.2% to 12% |
| Driver et al. (2020) |  | Stroke | Hospital  (rehabilitation ward) | *The GLB-CVA intervention* promotes self-management of a healthy lifestyle for people with CVD through individual engagement in health behavior change methods. The intervention is delivered in a group setting by a clinician lifestyle coach and includes: 1) education on the importance of healthy behaviors on heart health and stroke; 2) involving care partners in the sessions to provide physical and emotional support; 3) delivery of stroke-specific handouts on weight loss barriers and healthy lifestyle importance (e.g. physical activity and adaptive cooking); and 4) guest lectures by experts in the CVD field (e.g. physical therapists, dietitians). |  | RCT | 64 stroke patients | Study currently in progress |
| Hjelmfors et al. (2018) |  | Heart Failure | Heart failure clinic | The *Question Prompt List (QPL)* is a 7-page A4 booklet, containing 45 questions grouped into the topics: 1) heart failure and what to expect in the future; 2) help and support at deterioration; 3) end-of-life care issues; and 4) additional questions for the family members. It was designed to facilitate patient and carer communication about heart failure trajectory and end-of-life care with a healthcare professional. |  | Pre-post study | 13 cardiac healthcare professionals | Increase in self-reported knowledge, confidence, and skills of cardiac healthcare professionals. |
| Kjork et al. (2022) |  | Stroke | Community  (online) | *Strokehälsa™* is a pre-visit digital tool that includes well-validated self-report and health information to prepare people with stroke for a follow-up visit. It is based on the validated instrument Post-Stroke Checklist and the patient answers 14 questions and is offered information about health problems after a stroke. |  | - | - | - |
| Lalonde et al. (2014) |  | CVD | Primary care practice | The *TRANSIT program* comprises of six interventions: 1) the Quebec Health Ministry’s Internet-based directory of community and health resources to support self-management; 2) a protocol for interprofessional follow-up; 3) enhanced access to other health professionals; 4) collective prescriptions; 5) a patient health booklet; and 6) motivational interviewing. Primary care clinic nurses trained in motivational interviewing evaluate cardiovascular health and use the patient health booklet to support patients in setting a treatment plan. They refer patients to the most appropriate community and health resources, employing the electronic directory to provide patients and family members with accurate and pertinent information. Community pharmacists monitor medication adherence, safety, and efficacy. |  | - | - | - |
| Pekmezaris et al. (2016) |  | Heart Failure | Community  (telehealth) | A telemonitoring intervention that connects from the patient’s home via wireless transmission to the provider station (in both English and Spanish). The telemonitoring has intervention has two components: 1) a daily vital signs monitoring component (client-side operation), wherein patients monitor standard key indicators of possible condition exacerbation which are automatically transmitted to the server, and 2) a weekly telemonitoring face-to-face video visit, wherein patients attend a regularly scheduled tele-visit (real time) with a clinician. |  | - | - | - |
| Prick et al. (2022) |  | Stroke | Hospital | An online Patient Decision Aid with integrated outcome information for discharge planning of hospitalized patients with stroke to support patient education, clarification of patient values, and the process of shared decision making on discharge location and type of care after discharge from the hospital. It includes an interactive ‘patients-like-me’ model on the discharge location of comparable patients and a PROMs questionnaire on physical and mental well-being. |  | RCT | 630 stroke patients | Study currently in progress |
| Ramage et al. (2022) |  | Stroke | Community  (telehealth) | A 6-month telehealth-delivered exercise program delivered by a physiotherapist or exercise professional with experience in stroke care. The initial telehealth exercise session includes discussion of exercise and information delivery preferences; assessment of participant ability; and information/education regarding benefits of physical activity, discussion of program aims and strategies to optimise physical activity levels. Individually tailored, supervised, telehealth exercise sessions aim to include a wellness check; warm up; 20 minutes of moderate to vigorous physical activity; and a cool down. The program also includes therapist assistance or access to local volunteers or support services to support participants in accessing programs to continue physical activity. |  | RCT | 80 stroke patients | Study currently in progress |
| Raynor et al. (2020) |  | Heart Failure | Hospital (cariology ward) & Community pharmacies | The *Medicines At Transitions Intervention* is designed to optimize medicines management across a common ‘gap’ in care when patients are transferred between clinicians in different organizations. It comprises of five components: 1) A patient-held information resource covering information about heart failure medicines, their healthcare team, and symptoms, plus a checklist to complete detailing events that should have happened in hospital, at discharge, and after discharge; 2) transfer of discharge information to community pharmacy to encourage medicines reconciliation; and 3) an invitation to attend a post-discharge medication review with a community pharmacist. |  | - | - | - |
| Redfern et al. (2006) |  | Coronary Heart Disease | Hospital | A series of coronary heart disease risk factor modules and corresponding information leaflets for patients. The cholesterol-lowering module is a mandatory module for all patients, and the remaining modules for other CVD risk factors (blood pressure management, physical inactivity, smoking cessation) are optional. |  | - | - | - |
| Sabater-Hernandez et al. (2018) |  | Atrial Fibrillation | Pharmacy | A *Community Pharmacy Service* aimed at enhancing the management of atrial fibrillation, which includes patient education, self-monitoring of blood pressure/atrial fibrillation at home, and evaluation of results, referral, and follow-up. |  | - | - | - |
| Toledo-Chavarri et al. (2020) |  | Ischemic Heart Disease | Community  (online) | The *E-mpodera2 virtual community of practice (vCoP) intervention* is a virtual platform to interact and share tools/knowledge necessary for patient empowerment. Empowerment dimensions included health literacy, self-management, shared decision-making and social support. |  | - | - | - |
| Tongpeth et al. (2018) |  | ACS | Hospital (tablet/iPad) | Patient education via an Avatar app on a handheld tablet computer. The content of the Avatar app was divided into four sections: 1) heart attack warning signs quiz; 2) heart attack signs and symptoms; 3) what to do when having a heart attack; and 4) a Heart Attack Action Plan Quiz. To encourage engagement and reinforce learning, quizzes were placed at the beginning and the end of each section of the app. |  | RCT | 70 ACS patients | Knowledge, attitudes, and beliefs of heart attack symptoms significantly increased in intervention participants compared to controls at 6‐month follow-up. No differences between groups were observed in GP visits, ED visits, or 30‐day hospitalizations |
| Triantafyllidis et al. (2015) |  | Heart Failure | Community  (tablet/iPad) | A digital home monitoring system with an integrated risk prediction and disease management service, which provides tailored alerts and advice to patients and clinical decision support to healthcare practitioners (GPs, nurses, cardiologists). The system included an Android-based tablet computer that connected wirelessly via Bluetooth to a blood pressure and heart rate monitor and an electronic weighing scale. The application included additional features that allowed participants to review their personal readings via a graphical display, access educational material (such as video clips about heart failure and drug management), and to communicate with the study team (clinicians, administrators, engineers, and a social scientist). |  | RCT | 202 heart failure patients | No statistically significant between‐group differences were observed for treatment opportunity or HRQoL at 6‐month follow‐up |
| Walsh et al. (2019) |  | CVD | Community  (online) | *The PATHway intervention* is an e-health intervention for the self-management of CVD risk factors post-CR. PATHway uses an internet-enabled and sensor-based home exercise platform as the core component of a personalized, comprehensive lifestyle program. After completion of the center-based CR program, participants completed a cardiopulmonary exercise test. The results of the cardiopulmonary exercise test were used to determine the individual training heart rates, which were then entered into the PATHway system. Each participant was guided to train at a heart rate between their first and second ventilatory thresholds. Participants were encouraged to achieve the goal of 150 minutes of moderate intensity physical activity per week according to prevailing guidelines. Different exercise modalities (e.g. Exerclass, Exergame, Active lifestyle activity) were available to the participant. |  | RCT | 120 CVD patients | Average daily MVPA significantly increased in intervention participants compared to controls at 6‐month follow‐up.  Intervention participants maintained a stable CVD risk score, compared to controls.  No significant group differences regarding most physical fitness outcomes, HRQoL, exercise self-efficacy, medication adherence or diet at follow‐up. |
| Woods et al. (2018) |  | Heart failure | Community  (Smartphone app) | A consumer mHealth application to support people with heart failure to live well at home. The app has three main sections: 1) the Home Screen that provides a summary of patient goals and reminders; 2) the My Plan section that includes nine important components of self-management of heart failure (medications, symptoms, exercise, weight, fluid, well-being, diet, blood pressure and pulse, and future plans); and 3) A Health Management section that contains a medical documentation repository, appointment calendar, and health care professional contact details. |  | - | - | - |
| Zacharia et al. (2021) |  | Stroke | Community  (telehealth) | The *telehealth-delivered dietary (DIET) intervention* consists of 10 individual diet counselling sessions delivered over 6-months. The first two sessions focus on detailed initial assessment, program education, current diet history and assessment of barriers to change, the next five sessions aim to achieve high adherence to a Mediterranean diet pattern, and the final three sessions aim for self-efficacy in maintaining dietary change. |  | RCT | 80 stroke patients | Study currently in progress |

ACS=Acute coronary syndrome; CR=Cardiac Rehabilitation; CVD=Cardiovascular disease; ED=Emergency department; GP=General practitioner; HRQoL=Health-related quality of life; MVPA=Moderate to vigorous physical activities; RCT=Randomized controlled trial; - =Not applicable
