# Supplementary material for: Use of co‐design methodology in the development of cardiovascular disease secondary prevention interventions: A scoping review
Source: Health Expect. 2022 Nov 10;26(1):16–29. doi: 10.1111/hex.13633 (PMC9854329; doi:10.1111/hex.13633)
Supplement: Supplementary file 3 — Supplementary information. [file HEX-26--s002.docx]

**Supplementary File 3: Sufficiency of reporting of co-design methodology in the included studies**

| **Author (year)** | **Aim** | **Setting** | **Recruitment** | **Participants** | **Facilitators** | **Procedure** | **Schedule** | **Results** | **Intervention** | **Total**  **Score** |
| --- | --- | --- | --- | --- | --- | --- | --- | --- | --- | --- |
| Aaby et al.  (2020) | Yes | Yes | Yes | No | No | Yes | Yes | Yes | Yes | 7 |
| Ahmed et al. (2019) | Yes | Yes | Yes | Yes | Yes | Yes | Yes | Yes | Yes | 9 |
| Bonner et al. (2019) | Yes | Yes | No | Yes | No | Yes | Yes | Yes | Yes | 7 |
| Breeman et al. (2021) | Yes | No | No | Yes | No | Yes | Yes | Yes | Yes | 6 |
| Cornet et al. (2019) | Yes | Yes | Yes | No | Yes | Yes | Yes | No | Yes | 7 |
| Dorri et al.  (2021) | Yes | Yes | Yes | Yes | Yes | Yes | Yes | Yes | Yes | 9 |
| Driver et al.  (2020) | Yes | No | No | Yes | Yes | Yes | Yes | Yes | Yes | 7 |
| Hjelmfors et al. (2018) | Yes | No | Yes | Yes | Yes | Yes | Yes | Yes | Yes | 8 |
| Kjork et al. (2022) | Yes | Yes | Yes | Yes | No | Yes | Yes | Yes | Yes | 8 |
| Lalonde et al. (2012) | Yes | No | Yes | Yes | Yes | Yes | Yes | Yes | Yes | 8 |
| Pekmezaris et al. (2016) | Yes | No | Yes | No | Yes | Yes | Yes | Yes | Yes | 7 |
| Prick et al. (2022) | Yes | Yes | Yes | Yes | No | Yes | No | Yes | Yes | 7 |
| Ramage et al. (2022) | Yes | Yes | Yes | Yes | Yes | Yes | Yes | Yes | Yes | 9 |
| Raynor et al. (2020) | Yes | Yes | Yes | No | Yes | No | No | Yes | Yes | 6 |
| Redfern et al. (2006) | Yes | No | No | Yes | No | No | No | Yes | Yes | 4 |
| Sabater-Hernandez et al. (2018) | Yes | Yes | Yes | Yes | Yes | Yes | Yes | Yes | No | 8 |
| Toledo-Chavarri  et al. (2020) | Yes | Yes | Yes | Yes | Yes | Yes | Yes | Yes | Yes | 9 |
| Tongpeth et al. (2018) | Yes | Yes | Yes | Yes | Yes | Yes | Yes | Yes | Yes | 9 |
| Triantafyllidis  et al. (2015) | Yes | No | No | Yes | No | Yes | No | Yes | Yes | 5 |
| Walsh et al.  (2019) | Yes | No | Yes | Yes | No | Yes | No | Yes | Yes | 6 |
| Woods et al. (2018) | Yes | Yes | No | No | Yes | Yes | Yes | No | No | 5 |
| Zacharia et al. (2021) | Yes | No | Yes | No | No | Yes | No | Yes | Yes | 5 |
